# Daptomycin-Vancomycin–Resistant *Enterococcus faecium* Native Valve Endocarditis: Successfully Treated With Off-Label Quinupristin-Dalfopristin

**DOI:** 10.1177/2324709616665408

**Published:** 2016-09-15

**Authors:** Khandakar Hussain, Saad Ullah, Hassan Tahir, Waseem Zaid Alkilani, Muhammad Naeem, Nagadarshini Ramagiri Vinod, Samuel Massoud

**Affiliations:** 1 Temple University/Conemaugh Memorial Medical Center, Johnstown, PA, USA

**Keywords:** infective endocarditis, *Enterococcal faecalis*, quinupristin-dalfopristin, multidrug resistance, daptomycin-resistant enterococci

## Abstract

Multidrug-resistant enterococcal nosocomial invasive infections are a rising concern faced by the medical community. Not many options are available to treat these highly virulent organisms. Risk factors for developing these highly resistant organisms include prolonged hospital stay, previous antibiotic use, and immunosuppression. In this article, we report a case of daptomycin-resistant enterococcal native infective endocarditis treated with off-label use of quinupristin-dalfopristin.

## Introduction

Enterococcal species are known opportunistic pathogens that can lead to invasive infections in patients with prolonged antimicrobial use and hospitalizations.^1-4^ These organisms are the second most common nosocomial bloodstream pathogens isolated in the United States, with prevalence of vancomycin-resistant enterococci (VRE) currently exceeding 30% in intensive care units.^5^ There are limited effective antimicrobial treatment options for multidrug-resistant enterococcal infections. In this article, we describe a rare case of a patient with recurrent urinary tract infections (UTIs) who developed prolonged colonization with vancomycin-daptomycin–resistant *Enterococcal faecium* organisms leading to native mitral valve infective endocarditis. The patient was successfully treated with off-label use of quinupristin-dalfopristin (Q-D).

## Case Presentation

**Figure 1.**
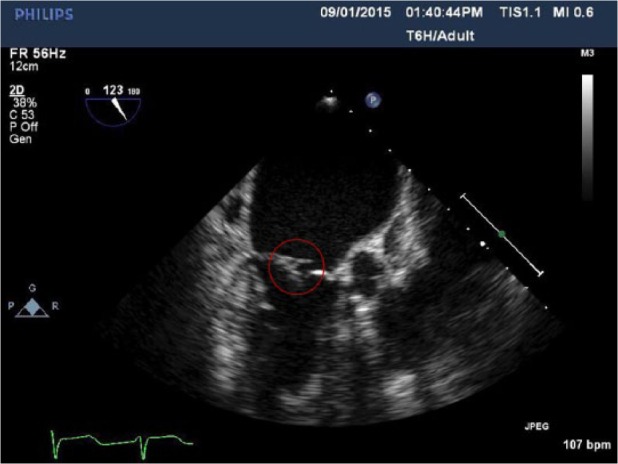
Encircled mitral valve vegetation.

**Figure 2.**
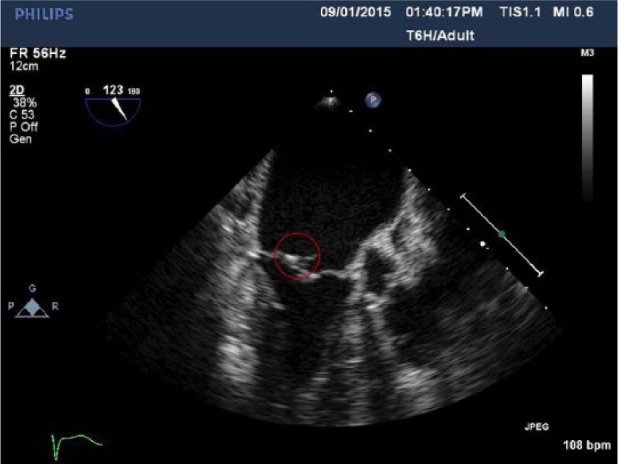
Another view of the mitral valve, showing mitral valve vegetation.

Our patient is an 87-year-old female with a past medical history significant for chronic kidney disease, hypertension, recurrent UTIs, severe mitral regurgitation, and atrial fibrillation who presented with a 3-day history of fever. Subsequently the patient was diagnosed with UTI secondary to VRE and was started on linezolid. The patient’s blood cultures were consistently positive despite being on linezolid. This raised suspicion for other possible sources of infection. Transesophageal echocardiogram was done and a 7 mm × 2 mm mobile structure attached to the mitral valve consistent with mitral valve vegetation with severe eccentric mitral regurgitation directed anteriorly was discovered (Figures 1 and 2). Repeat cultures grew daptomycin-vancomycin–resistant *Enterococcus faecium*. The organism’s sensitivity was intermediate to linezolid (minimum inhibitory concentration [MIC] 4), and resistant to vancomycin (MIC >32) and daptomycin (MIC 6). Cardiothoracic surgery was consulted for a possible valve surgery, but the patient refused surgical intervention. Monotherapy with intravenous Q-D was started and the patient showed signs of clinical improvement. Subsequent blood cultures were negative, and the patient was discharged on a 6-week course of intravenous antibiotic. The patient remained clinically stable on further follow-up after 8 weeks with no evidence of vegetation on repeat echocardiogram.

## Discussion

Enterococcal infections are second only to staphylococci as a cause of gram-positive nosocomial infections.^2^ These organisms have an extraordinary ability to acquire antibiotic resistance. The increase in the antimicrobial resistance against these organisms poses a huge challenge for physicians while treating these pathogens. Risk factors for vancomycin-resistant enterococcal infections include prolonged hospitalization, high APACHE II score, previous exposure to antibiotics, repeated gastrointestinal surgeries, immunosuppression, organ transplantation, and malignancies.^4^ Variety of antibiotic resistance mechanisms have been described including alteration of drug binding sites and inactivation of antimicrobial agents that promotes survival in the human host.^2^ Because of the increased prevalence of multidrug-resistant *Enterococcus faecium*, there has been major decrease in treatment options as majority of the *Enterococcus faecium* isolates are now resistant to ampicillin, vancomycin, and aminoglycosides,^6^ which are three of the commonly used antibiotics against enterococcal infections. Antibiotics such as linezolid, daptomycin, and tigecycline have shown reliable in vitro activity against enterococcal bacteria.^2^

Daptomycin is the only Food and Drug Administration (FDA)–approved cyclic lipopeptide antibiotic with in vitro bactericidal activity against VRE. Daptomycin alone for the treatment of VRE endocarditis is not recommended due to high resistance.^5^ Daptomycin resistance has been reported with invasive infections including osteomyelitis, prosthetic devices, and endocarditis at doses of ≤6 mg/kg.^5^ Not much has been studied about the frequency of daptomycin nonsusceptibility enterococcal species.^3^ Linezolid is also an FDA-approved bacteriostatic antibiotic for treatment in this scenario, but treatment rates remain unreliable and resistance rates of 20% have been reported.^5^ In our case, sensitivity was intermediate to linezolid. Therefore, it was not used. Tigecycline has also been used for the treatment of vancomycin-resistant *Enterococcus faecium* infections but without much success.^5^

Q-D is a combination of parenteral streptogramin antibiotics in 30:70 ratio that inhibits bacterial protein synthesis by binding to prokaryotic ribosomes. These antibiotics have in vitro activity against gram-positive organisms including *Streptococci, Staphylococci*, and vancomycin-resistant *Enterococcus faecium* with an MIC of 1 µg/mL (except for *Enterococcus faecalis*).^7^ The efficacy and safety of Q-D was evaluated in 396 patients where no alternative antibiotic was available in a prospective multicenter study for the treatment of vancomycin-resistant *Enterococcus faecium* infections with an overall clinical response observed in 65.6% of the patients. However, the study did not include patients with infective endocarditis. Arthralgia and myalgia were the most common reversible adverse events with Q-D reported in the same study.^7^ A study by Linden et al in 1997 found that there was less recurrence of bacteremia, less persistence of infection at the primary site, and lower mortality in vancomycin-resistant *Enterococcus faecium* bacteremic patient treated with Q-D.^8^

Our patient had daptomycin-vancomycin–resistant *Enterococcus faecium* native mitral valve endocarditis that was successfully treated with parenteral Q-D. Q-D is approved by FDA for complicated skin and soft tissue infections^9^ and serious or life-threatening vancomycin-resistant *Enterococcus faecium* bacteremia. However, its use is not recommended for endocarditis though varying results have been reported with Q-D.^10^ Similarly, tigecycline has been used with variable success in patients with vancomycin-resistant *Enterococcus faecium* native mitral valve endocarditis.^10^ Our patient was treated with off label use of Q-D with good response as evident by the resolution of bacteremia on repeat blood cultures at 4 days posttreatment and improvement in the patient’s clinical condition. This pharmacological approach can be of benefit in improving patient outcomes when other options cannot be used reliably.

## Conclusion

Physicians should be aware of the emergence of highly resistant enterococcal species and should monitor MICs of organisms isolated during treatment.
